# Next-Generation Sequencing reveals relationship between the larval microbiome and food substrate in the polyphagous Queensland fruit fly

**DOI:** 10.1038/s41598-019-50602-5

**Published:** 2019-10-01

**Authors:** Rajib Majumder, Brodie Sutcliffe, Phillip W. Taylor, Toni A. Chapman

**Affiliations:** 1 Department of Biological Sciences, Macquarie University, North Ryde, NSW 2109 Australia; 2 Department of Environmental Sciences, Macquarie University, North Ryde, NSW 2109 Australia; 3 Biosecurity and Food Safety, NSW Department of Primary Industries, Elizabeth Macarthur Agricultural Institute (EMAI), Menangle, NSW 2567 Australia

**Keywords:** Microbiology, Microbiome

## Abstract

Insects typically host substantial microbial communities (the ‘microbiome’) that can serve as a vital source of nutrients and also acts as a modulator of immune function. While recent studies have shown that diet is an important influence on the gut microbiome, very little is known about the dynamics underpinning microbial acquisition from natural food sources. Here, we addressed this gap by comparing the microbiome of larvae of the polyphagous fruit fly *Bactrocera tryoni* (‘Queensland fruit fly’) that were collected from five different fruit types (sapodilla [from two different localities], hog plum, pomegranate, green apple, and quince) from North-east to South-east Australia. Using Next-Generation Sequencing on the Illumina MiSeq platform, we addressed two questions: (1) what bacterial communities are available to *B. tryoni* larvae from different host fruit; and (2) how does the microbiome vary between *B. tryoni* larvae and its host fruit? The abundant bacterial taxa were similar for *B. tryoni* larvae from different fruit despite significant differences in the overall microbial community compositions. Our study suggests that the bacterial community structure of *B. tryoni* larvae is related less to the host fruit (diet) microbiome and more to vertical transfer of the microbiome during egg laying. Our findings also suggest that geographic location may play a quite limited role in structuring of larval microbiomes. This is the first study to use Next-Generation Sequencing to analyze the microbiome of *B. tryoni* larvae together with the host fruit, an approach that has enabled greatly increased resolution of relationships between the insect’s microbiome and that of the surrounding host tissues.

## Introduction

Insects commonly have close relationships with a diverse microbiome that has substantial influence on their ecology and evolution through immunity development, pathogen resistance, gut physiology and fitness at every stage of the life cycle^1–5^. These relationships may be beneficial or harmful to the host health and fitness, depending on the composition of the microbiome^6–9^. Symbiotic and endosymbiotic bacteria can serve as an important source of essential nutrients to their host insects^10–12^ and enhance resistance against pathogens, plant defences or pesticides^13–17^. Insect microbial communities often have a positive influence on egg maturation and production, physiological development and survival^2,18,19^.

The existence of a symbiotic relationship between tephritid fruit flies and their microbiome has been known for almost 100 years^20,21^. As a prominent example, bacterial symbionts of *Bactrocera oleae* (olive fruit fly) play a vital role in the digestion of green olive, specifically protein hydrolysis^22^. *Candidatus Erwinia dacicola* in the larval microbiome of *B. oleae* provides essential amino acids and enables the larvae to develop in unripe olive that contain oleuropein, which inhibits development of other insects^23^. *Candidatus Erwinia dacicola* also increases reproduction in *B. oleae*^24^. The community of nitrogen fixing bacteria (e.g. Enterobacteriaceae) improves development and reproduction in *Ceratitis capitata* (Mediterranean fruit fly, or ‘medfly’)^10^. Numerous studies have demonstrated that gut bacteria are associated with digestion, detoxification, immune response, metabolism, sexual behaviour, reproduction and survival in tephritid flies^18,23,25–29^.

*Bactrocera tryoni* (Queensland fruit fly, or ‘Q-fly’) is a highly polyphagous tephritid fly that is widespread along the east coast of Australia where it is a significant pest of horticulture^30–32^. The most common gut bacterial families identified in *B. tryoni* include Enterobacteriaceae, Acetobacteraceae, Streptococcaceae, and Enterococcaceae^33–36^. These bacteria families are also common in other polyphagous fruit flies, including *B. neohumeralis*, *B. carambolae, B. jarvisi*, and *C. capitata*^9,34,36–38^. Several studies have investigated the bacterial communities of *B. tryoni* larvae and adults, providing partial identification of gut microbes^34,38,39^. A recent study of *B. tryoni* larvae used near full-length 16S analysis as a proof-of-concept study investigating the bacterial populations in the midgut from one type of fruit from different two locations^39^. In addition, pyrosequencing^34,40^ and culture dependent methods have been applied to evaluate *B. tryoni* gut bacterial identifications^39^. Experimental techniques and conditions may influence the results of culture-dependent methods^41^ and the biases and sampling limitations of techniques used to date to identify microbial communities in *B. tryoni* are well-documented^34^. With the advent of next-generation sequencing techniques we are now able to overcome these technical issues for a more comprehensive investigation of the *B. tryoni* microbial communities^39^.

Despite clear evidence that the microbiome is a major mediator of fitness in tephritid flies^36,40,42–44^, substantial knowledge gaps remain in the physiological and ecological diversity of the *B. tryoni* gut microbial community^34,38,39^. These knowledge gaps include how *B. tryoni* larvae acquire their microbial community and the ecological interaction between fruit hosts and *B. tryoni* larvae in nature. In the present study, we (i) comprehensively investigate the microbiome of wild *B. tryoni* larvae from a range of fruits that have been infested in nature, (ii) explore the effect of fruit host on structure of *B. tryoni* microbial communities, and (iii) assess the role of vertical transfer structuring these microbial communities. We profiled larval microbial communities by sequencing the 16S ribosomal RNA (rRNA) gene from whole insects using Next-Generation Sequencing (NGS). This technique is ideal for identifying the majority of cultivable and uncultivable microbes and, along with our sampling of multiple host fruit, enables the most comprehensive survey of *B. tryoni* microbial communities to date.

## Results

### Identification of wild larvae as *B. tryoni*

Sanger sequencing of the COI gene confirmed that all 36 wild larvae, collected from 5 different fruit type/origins were *B. tryoni*. Additionally, ~600 adult flies obtained from the collected fruits and were identified as *B. tryoni* by morphology. No other fly species was identified from the experimental samples.

### Profile of *B. tryoni* larval microbiome

A total of 167 bacterial OTUs were detected in *B. tryoni* larvae. These represented 8 phyla, 18 classes, 27 orders, 53 families and 78 genera (Supplementary Data S1, S2). Despite this broad taxonomic range, the majority of these taxa were rare in abundance; only 16 OTUs (~5%) were classed as abundant, i.e. representing ≥1% of the microbiome in one or more larvae (Table 1). Further, an average of 97% of the larval microbiome was made up of proteobacterial taxa.

**Table 1:** Taxonomic identification of the of the 16 most abundant bacterial OTUs in the larvae and fruit.

OTU ID	%Fruit	%Larvae	Phylum	Class	Oder	Family	Genus	Species
OTU_1	15.2%	53.1%	Proteobacteria	Alphaproteobacteria	Rhodospirillales	Acetobacteraceae	*Swaminathania/Asaia*	
OTU_3	35.2%	7.0%	Proteobacteria	Alphaproteobacteria	Rhodospirillales	Acetobacteraceae	*Gluconobacter*	
OTU_2	28.5%	9.2%	Proteobacteria	Alphaproteobacteria	Rhodospirillales	Acetobacteraceae	*Gluconacetobacter*	*intermedius*
OTU_6	4.9%	2.0%	Firmicutes	Bacilli	Lactobacillales	Leuconostocaceae	*Leuconostoc*	
OTU_5	1.4%	5.3%	Proteobacteria	Gammaproteobacteria	Enterobacteriales	Enterobacteriaceae	*Tatumella*	
OTU_368	3.6%	2.6%	Proteobacteria	Alphaproteobacteria	Rhodospirillales	Acetobacteraceae	*Acetobacter*	
OTU_7	0.0%	5.9%	Proteobacteria	Gammaproteobacteria	Enterobacteriales	Enterobacteriaceae		
OTU_70	0.1%	5.1%	Proteobacteria	Gammaproteobacteria	Enterobacteriales	Enterobacteriaceae	*Klebsiella*	*oxytoca*
OTU_92	2.4%	1.9%	Proteobacteria	Alphaproteobacteria	Rhodospirillales	Acetobacteraceae		
OTU_8	2.6%	1.0%	Proteobacteria	Alphaproteobacteria	Rhodospirillales	Acetobacteraceae	*Acetobacter*	
OTU_53	2.7%	0.4%	Proteobacteria	Alphaproteobacteria	Rhodospirillales	Acetobacteraceae		
OTU_11	0.0%	2.8%	Proteobacteria	Gammaproteobacteria	Enterobacteriales	Enterobacteriaceae	*Providencia*	
OTU_10	0.0%	1.4%	Proteobacteria	Gammaproteobacteria	Enterobacteriales	Enterobacteriaceae		
OTU_9	0.5%	0.9%	Proteobacteria	Gammaproteobacteria	Oceanospirillales	Halomonadaceae		
OTU_174	0.6%	0.1%	Proteobacteria	Alphaproteobacteria	Rhodospirillales	Acetobacteraceae		
OTU_13	0.5%	0.2%	Proteobacteria	Gammaproteobacteria	Xanthomonadales	Xanthomonadaceae	*Frateuria*	*aurantia*

**Figure 1:**
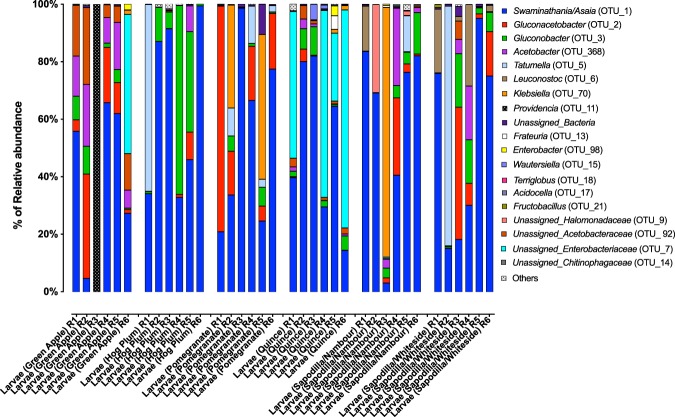
Relative abundance of gut bacterial taxa of *B. tryoni* wild larvae (genus level). The percentage of relative abundance of four or less than are included in “Others”. Six wild larvae from five different types of fruits are plotted and R1 to R6 refers to the replicate number of each fruits.

**Figure 2:**
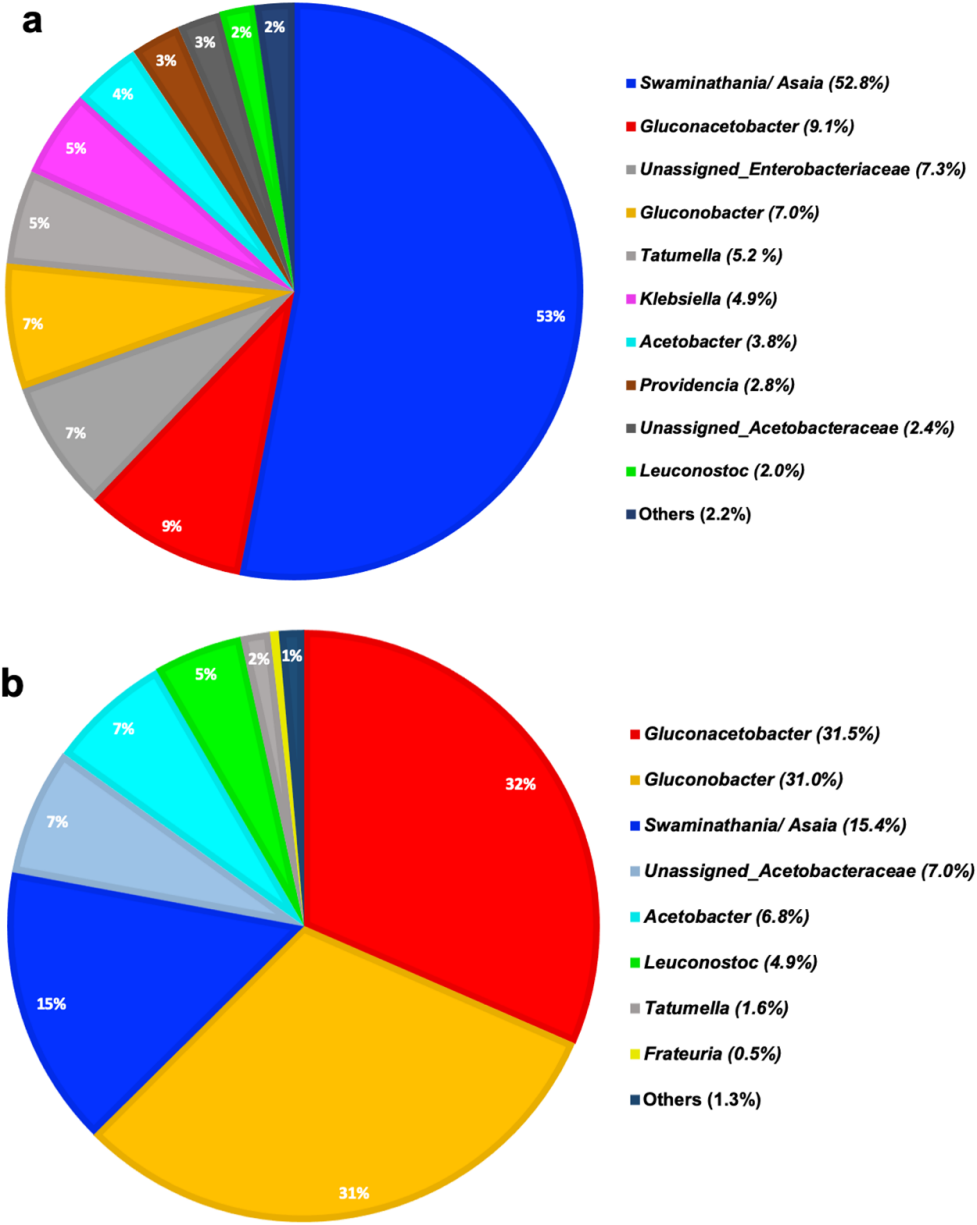
Percentage of mean relative abundance of the bacteria at the genus levels in (**a**) the *B. tryoni* wild larval samples and (**b**) fruit samples.

The majority of detected proteobacterial taxa belonged to just two families. The alphaproteobacterial *Acetobacteraceae* represented an average of 75% of the larval microbiome, and the Gammaproteobacterial Enterobacteriaceae represented an additional 21% (Supplementary Fig. 1). The next most abundant family was the Leuconostocaceae, from the phylum Firmicutes, which had an average relative abundance of 2% (Supplementary Fig. 1). Leuconostocaceae taxa represented ≥1% of microbiome in only 5 of the 36 larvae, and thus were only sporadically abundant. This contrasts with the alphaproteobacterial Acetobacteraceae and Gammaproteobacterial Enterobacteriaceae taxa, which represented ≥1% of the larval microbiome in 35 and 14 of 36 larvae, respectively. At a finer taxonomic resolution, the genus *Swaminathania/Asaia* constituted more than 50% of the larval microbiome (53%) and was abundant in 35 of 36 larvae samples (Fig. 1). Other genera representing an average abundance of ≥1% of the larval microbiome included *Gluconacetobacter* (9.1%), *Gluconobacter* (7%), *Tatumella* (5.2%), *Klebsiella* (4.9%), *Acetobacter* (3.8%), *Providencia* (2.8%) and *Leuconostoc* (2%) (Fig. 1, Fig. 2a). These genera, however, were abundant in <50% of the sampled larvae (Fig. 2a).

### Larvae from different fruit types have different microbiomes

**Figure 3:**
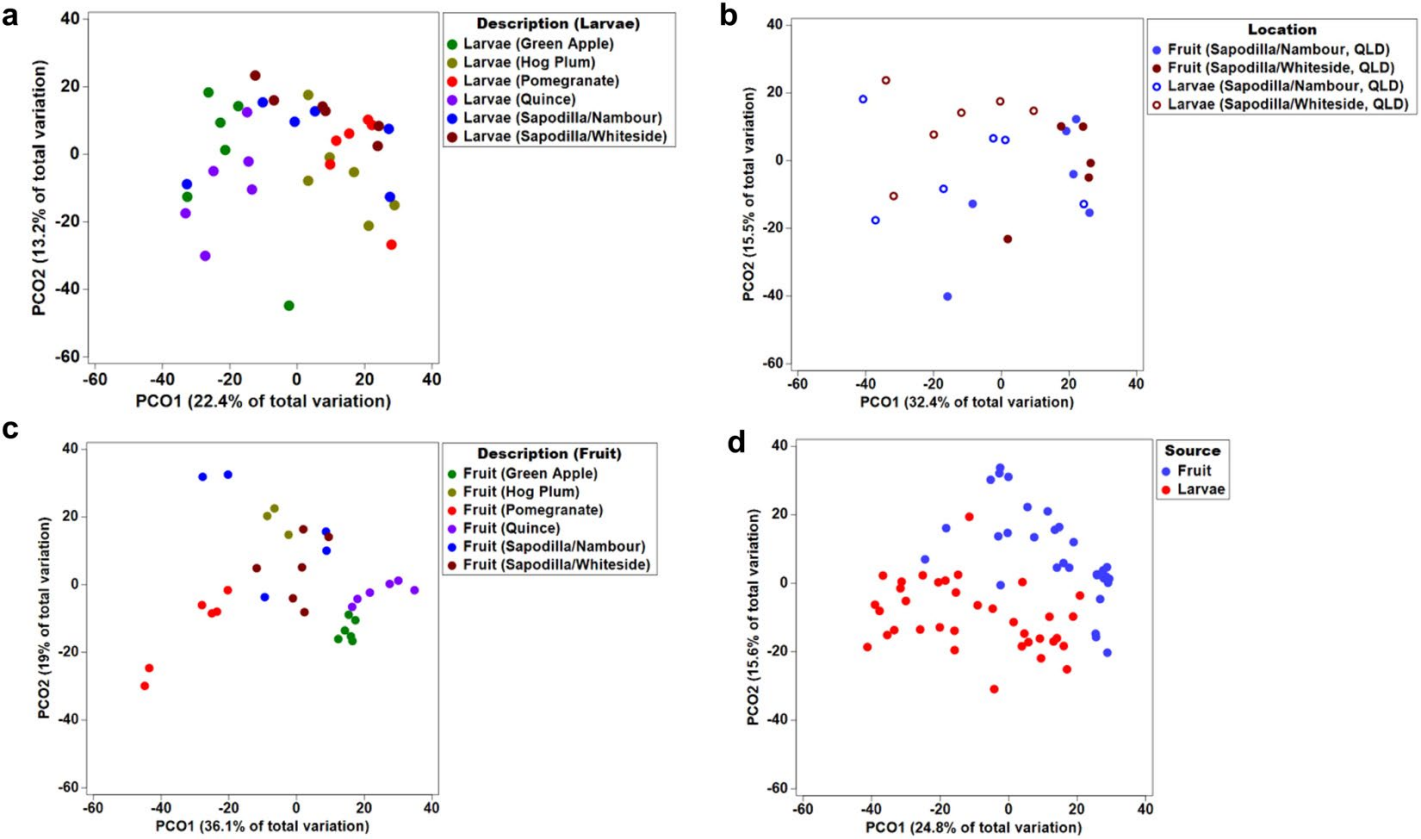
Principal co-ordinate analysis (**a**) the larval gut bacteria of *B. tryoni* from five type of fruit sources*;* (**b**) Bacterial community composition of the *B. tryoni* larvae collected from 2 different location (Sapodilla); (**c**) Bacterial community composition in the five different fruit; (**d**) bacterial population between larvae and fruit. Different color point indicates different fruit type and the larvae respectively.

The microbiome of *B. tryoni* larvae varied among different types of fruit (Fig. 3a). While alphaproteobacterial Acetobacteraceae and Gammaproteobacterial Enterobacteriaceae were the most dominant bacterial families in *B. tryoni* larvae, overall (Supplementary Fig. 1a), the relative abundance of Unassigned Acetobacteraceae and Unassigned Enterobacteriaceae were found to differ significantly (FDR corrected, P < 0.01 and P < 0.05, respectively) between different types of fruit (Supplementary Data S3). *Acetobacter* (P < 0.05) was associated with the larval microbiome from all the types of fruit source except pomegranate. On the other hand, *Providencia* was observed only in one green apple larval sample (replicate 3) (Fig. 1). In addition, we only detected Bacilli (2%) in larvae from sapodilla fruit collected from Nambour, QLD and Whiteside, QLD. Further, only larvae from quince contained *Flavobacteria* (1%) (Fig. 1). Leuconostocaceae and Halomonadaceae were abundant in several larvae from sapodilla. Genus level relative abundance of *Leuconostoc* (P < 0.001)*, Staphylococcus* (P < 0.001) and *Terriglobus* (P < 0.01) were significantly different in larvae from the five different fruits host. In addition, principal coordinate analysis (PCoA) of the microbial community structure of *B. tryoni* larvae from different fruits showed that larval microbial composition from green apple and quince were closer in the ordination plot than the other fruits (Fig. 3a).

### Microbial communities in fruit samples

In parallel with larval microbiome analysis*,* samples of the fruit tissues (fruit flesh) from which these larvae were collected, were analyzed for their bacterial microbiome communities. A total of 66 bacterial taxa were detected in fruit samples. 32 fruit samples had sufficient sequencing depth to be included in the current study (>10,000 reads). Of the four samples with fewer reads which were excluded from the study, three were of hog plum and one was of sapodilla from Nambour, QLD. Bacterial taxa detected in fruits represented seven phyla, twenty-seven families and thirty-six genera (Supplementary Data S1, S2). As in the larval microbiome communities, phyla Proteobacteria and Firmicutes were the most abundant, comprising 99.8% of fruit flesh microbial communities. Proteobacteria represented ≥95% of the communities in all fruit samples tested. We detected 4 families with high average relative abundances in fruit samples; Acetobacteraceae (92%), Leuconostocaceae (5%), Enterobacteriaceae (1.8%) and Halomonadaceae (0.5%) (Supplementary Fig. 1b). At the genus level, only seven taxa had an average relative abundance of >1%. These were *Gluconacetobacter* (35.5%), *Gluconobacter* (28.5%), *Swaminathania/Asaia* (15.2%), *Acetobacter* (3.8%), Unassigned Acetobacteraceae (6.3%), *Leuconostoc* (4.9%), and *Tatumella* (1.4%) (Fig. 2b, Supplementary Fig. 2).

The relative abundance of *Gluconobacter* was the highest at 89.1% in hog Plum (Supplementary Fig. 2). This relative abundance was significantly greater than that found in the other fruits (FDR corrected P < 0.0001). Similarly, no significant difference of the relative abundance of *Gluconacetobacter* and *Gluconobacter* were observed among green apple, quince, and sapodilla (two locations). Unassigned Acetobacteraceae (FDR corrected P < 0.0001), Unassigned Enterobacteriaceae (FDR corrected P < 0.05), *Acetobacter* (FDR corrected P < 0.05), *Frateuria* (FDR corrected P < 0.05) and *Swaminathania/Asaia* (FDR corrected *P < 0.05*) were all significantly difference among fruit types (Supplementary Data S3). A close observation of the microbial community structure of the different fruit types evaluated by the principal coordinate analysis (PCoA) found significant different in microbial composition between fruit types. However, the ordination plot showed that the microbial composition of the few samples from green apple and quince were close and overlapped (Fig. 3c)

### Larval microbiomes differ from those found in fruits

**Figure 4:**
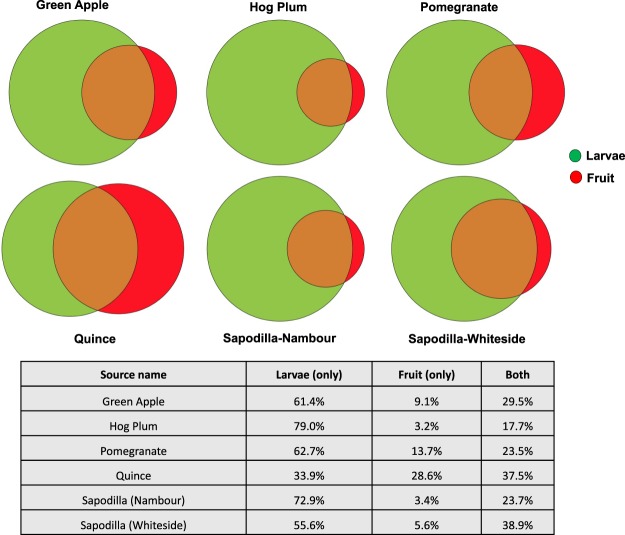
Venn diagram of the percentage of the bacteria present in the larvae only, fruits only and common in both collected from five different types of fruit in the wild.

Biodiversity metrics, including total species, species richness, Pielou’s evenness, Shannon’s and Simpson’s biodiversity indices, did not differ significantly between larval microbiomes and fruit (Supplementary Table 1). However, the Venn diagram showed that the average percentage of unique bacteria present only in larvae (61.7%) was much higher than the average percentage of unique bacteria present only in fruits (10.7%) (Supplementary Fig. 3). The percentage of bacteria present both in larvae and fruits was significantly higher than the bacteria found only in the fruits except in Quince (Fig. 4). The composition of the larval microbiome was significantly different from respective fruit flesh sample communities (PERMANOVA < 0.05, Table 2). This was reflected in the separation of the larval microbiome and bacterial community in the fruit flesh samples in the ordination plot (Fig. 3d). A number of differences in the relative abundance of abundant taxa were observed when comparing larval microbiomes with fruit flesh microbial communities. *Swaminathania/Asaia* was significantly more abundant in larvae compared to the fruits (FDR corrected P < 0.0001), while the opposite was observed for *Gluconacetobacter* (FDR corrected P < 0.05) and *Gluconobacter* (FDR corrected P < 0.001) (Fig. 2a,b, Supplementary Data S3). Surprisingly, two abundant bacterial genera, *Tatumella* and *Klebsiella*, were commonly observed in larvae but were rare in fruit (Fig. 2a,b). Unassigned bacteria (FDR corrected P < 0.001) and Unassigned proteobacteria (FDR corrected P < 0.0001) were found to be significantly different in relative abundance among fruits and larvae.

**Table 2:** PERMANOVA test (P values) from Pair-wise tests to compare the variation of the bacterial community between five different fru it and their larvae (*B. tryoni*).

	Fruit (Green Apple)	Fruit (Hog Plum)	Fruit (Pomegranate)	Fruit (Quince)	Fruit (Sapodilla/Nambour)	Fruit (Sapodilla/Whiteside)	Larvae (Green Apple)	Larvae (Hog Plum)	Larvae (Pomegranate)	Larvae (Quince)	Larvae (Sapodilla/Nambour)	Larvae (Sapodilla/Whiteside)
**Fruit (Green Apple)**												
**Fruit (Hog Plum)**	0.012											
**Fruit (Pomegranate)**	0.004	0.012										
**Fruit (Quince)**	0.001	0.012	0.002									
**Fruit (Sapodilla/Nambour)**	0.003	0.111	0.003	0.002								
**Fruit (Sapodilla/Whiteside)**	0.003	0.04	0.003	0.004	0.174							
**Larvae (Green Apple)**	0.003	0.017	0.001	0.003	0.011	0.006						
**Larvae (Hog Plum)**	0.002	0.015	0.002	0.005	0.005	0.006	0.003					
**Larvae (Pomegranate)**	0.003	0.015	0.002	0.007	0.003	0.002	0.003	0.007				
**Larvae (Quince)**	0.001	0.007	0.002	0.009	0.006	0.006	0.059	0.002	0.004			
**Larvae (Sapodilla/Nambour)**	0.005	0.028	0.002	0.002	0.086	0.004	0.028	0.044	0.007	0.02		
**Larvae (Sapodilla/Whiteside)**	0.002	0.013	0.002	0.002	0.004	0.004	0.004	0.004	0.006	0.001	0.483	

### Geographic location did not influence larval microbiome

Principal coordinate analysis and PERMANOVA tests both indicated that bacteria in sapodilla fruits and larvae did not differ between geographic locations (PERMANOVA test, fruit P = 0.151, larvae P = 0.094; Fig. 3b). Otherwise, the microbial community differed significantly among fruit types (Table 2, Fig. 3c). In contrast, PERMANOVA analysis indicated that microbiome of *B. tryoni* larvae from different fruit sources had similar bacterial composition except for those from pomegranate (Table 2). However, significant variation in microbiome was observed between larvae from the same fruits (Table 2, Fig. 3a).

## Discussion

This study presents comprehensive data on the microbiome of *B. tryoni* larvae collected from fruits that were infested in nature. By sampling from five different fruit types, we are able to explore meaningful ecological questions regarding the effect of host fruit on microbiome communities, while the inclusion of fruit flesh samples allowed us to explore the role of horizontal transfer in the microbial colonization. This is the first microbial survey to assess the microbiome of *B. tryoni* larvae from different types of fruits together with parallel assessment of the host fruit microbial community. We found that the larval microbiome composition differed substantially from the microbial community of the fruit that larvae were obtained from. Our findings suggest that the larval gut acts as a strong environmental filter, and while there was overlap in microbial community of the fruit and larvae, taxa that were abundant in fruit were not necessarily abundant in the larvae. Despite substantial variation in the microbial community of individual *B. tryoni* larvae, the most abundant taxa in the larvae were consistent across the different fruit sources. Thus, the differences detected in PERMANOVA were driven by low abundance taxa within the larval microbiome. Our study suggests that the microbial communities inside the fruits strongly influence the structure of bacterial communities present in the *B. tryoni* larvae.

Analysis of the microbiome of *B. tryoni* larvae revealed that the bacterial community in the larvae was dominated by one phylum, Proteobacteria, with 97% of the total sequences assigned to these taxa. Phyla Proteobacteria and Firmicutes have previously been reported as common in the midgut of *B. tryoni* larvae collected from peach fruits in the field and in domesticated colonies^39^, as well as in other fruit flies^40,45^ and in other insects, including butterflies^46^ and mosquitoes^47^.

Seventy five percent of average bacterial relative abundance was from the family of alphaproteobacterial Acetobacteraceae. Fruits are an abundant source of sugar, and insects emerging from fruits commonly host acetic acid bacteria^48^. We observed a high abundance of five bacterial genera - *Swaminathania/Asaia, Gluconacetobacter, Gluconobacter, Acidocella*, and *Acetobacter* - among others in the larval microbiome. Other studies have also observed Acetobacteraceae in *B. oleae*^49^, *Apis mellifera mellifera* (Hymenoptera: Apidae) (Honeybee)^50,51^, *Saccharococcus sacchari (*Cockerell) (Homoptera: Pseudococcidae) (Pink sugar cane mealybug)^52^, and *Drosophila*^53^. The alphaproteobacterial Acetobacteraceae helps to break down and digest complex glucose structure and lipid content present in larval diet^54^. *Swaminathania/Asaia* and *Acetobacter* are the two key bacteria commonly found in the gut of insects^26,48^. Previous studies have found that *Acetobacter pomorum* and *Swaminathania/Asaia* provide nutrients that improve larval development of *Drosophila* and *Anopheles gambiae* (Mosquito)^55,56^. Shin *et al*.^56^ demonstrated that *A. pomorum* in *Drosophila melanogaster* stimulates insulin growth factor signaling to maintain metabolic homeostasis and physiological development by pyrroloquinoline quinone-dependent alcohol dehydrogenase (PQQ-ADH) activation. The addition of acetic acid as a metabolic product of PQQ-ADH in the diet may improve the metabolic homeostasis of flies. Previous studies report that *Swaminathania/Asaia* sp. produces acetic acid which is involved in nitrogen-fixing and improves metabolic homeostasis of *B. tryoni*^39,57^. Surprisingly, low populations of *Swaminathania/Asaia* were observed in wild *B. tryoni* adults^34^, as well as in *B. oleae*^58^. In our study, the high dominance of *Swaminathania/Asaia* of 52.9% average relative abundance was observed in the presence of *Gluconacetobacter* and *Gluconobacter* in all 36 larvae samples. This suggests that Acetobacteraceae plays an important role in *B. tryoni* larvae.

The family of Gammaproteobacterial Enterobacteriaceae had an average relative abundance of 20.5% in *B. tryoni* larvae. *Enterobacter, Klebsiella*, and *Taumella*, are all major bacterial genera (average relative abundance > 0.1). From mammals to insects, Gammaproteobacterial Enterobacteriaceae commonly have mutualistic relationships in the host gut^26,59^. *Enterobacter* and *Klebsiella* have been reported in four *Bactrocera* species - *B. tryoni, B. neohumeralis, B. jarvisi*, and *B. cacuminata* - by using both pyrosequencing^34^ and culture dependent methods^60^. Deutscher *et al*.^39^ suggested that Gammaproteobacterial Enterobacteriaceae are crucial for survival of the larvae, transmitted vertically in *B. tryoni* and other tephritid. Further, these bacteria enhance metabolic activities in *C. capitata* and *B. oleae* larvae to support nitrogen fixation and pectinolysis^24,61^. Previous study also found Gammaproteobacterial Enterobacteriaceae enable the host *B. oleae* larvae to survive inside unripe olive fruits^24^. In *B. oleae* and *C. capitata* mass rearing programs using artificial larval diets, strains of Gammaproteobacterial Enterobacteriaceae have been added to the diet to improve pupal weight and mating performance, and decrease developmental time^62–66^. In contrast to beneficial bacteria, pathogens have also been reported including *Providencia* which is able to cause infection in *C. capitata*^67^ and *D. melanogaster*^68^. *Providencia*, a gram-negative opportunistic, non-spore forming pathogen^69^, has been observed and isolated from many other insects including *Lucilia sericata* (Diptera: Calliphoridae) (Blow fly)^70^, *Stomoxys calcitrans* L. (Diptera: Muscidae) (Stable fly)^71^, *Anastrepha ludens* (Diptera: Tephritidae) (Mexican fruit fly)^72^ and *B. oleae*^49^. In *D. melanogaster*, a strain of *Providencia*, *P. sneebia*, caused host mortality because of its ability to avoid detection by the insect host’s immune system^68^. In the present study, *Providencia* was found only in one larva from a single green apple (Fig. 1).

The family Leuconostocaceae was observed in *B. tryoni* larvae along with the genera *Leuconostoc* and *Fructobacillus*. Although not many studies have reported lactic acid bacteria in insects, especially in tephritid, *Leuconostoc* has also been reported in adults of other tephritid including *C. capitata, B. neohumeralis, B. tryoni* and *B. cacuminata*^34,38,42^. Furthermore, in *Drosophila, Leuconostoc* has been identified in both wild and domesticated populations^73,74^. In our analysis, *Leuconostoc* was observed in all of the larval samples collected from sapodilla in Nambour, QLD and in Whiteside, QLD. In contrast, *Fructobacillus* was only observed in the larvae collected from sapodilla in Whiteside, QLD. This finding suggested that the *Leuconostoc* may be transmitted horizontally from the sapodilla fruit to the larval gut. We also found Weeksellaceae, Xanthomonadaceae, Halomonadaceae, Acidobacteriaceae, and Chitinophagaceae in some larvae samples. Previous studies detected Xanthomonadaceae in soil and plant samples^75,76^. Weeksellaceae has been found to be a dominant element of the microbiome in *B. carambolae* larvae and pupae^36^ and *Colaphellus bowringi* (Coleoptera: Chrysomelidae) (Cabbage beetle)^28^.

The bacterial community in green apple, hog plum, pomegranate, quince, sapodilla (Nambour, QLD) and sapodilla (Whiteside, QLD) was not different in total bacterial load or species richness, but differed in composition, which is not unexpected given the fruit composition. The genera *Gluconacetobacter* (35.5%), *Gluconobacter* (28.5%), *Swaminathania/Asaia* (15.2%), *Leuconostoc* (4.9%), *Acetobacter* (3.8%) and *Tatumella* (1.4%) were observed in all of the fruit samples. The microbial communities in host fruits depends on a wide diversity of influences including fruit physiology, larval density, and environment conditions^53,77^. Previous studies reported that female tephritid fruit flies (e.g. *B. oleae* & *C. capitata*) inoculate fruit with bacteria from the family of Gammaproteobacterial Enterobacteriaceae during oviposition^42,58,78^. In our investigation, only a few bacterial families dominated including Acetobacteraceae (91.9%), Leuconostocaceae (5%), Enterobacteriaceae (1.8%) and Halomonadaceae (0.5%). The infestation by *B. tryoni* and the over-ripe status of the fruit might be why we observed 91.9% of the average relative abundance of the Acetobacteraceae and its associated bacterial genera of *Gluconacetobacter* (35.5%), *Gluconobacter* (28.5%) and *Swaminathania/Asaia* (15.2%). The variation of bacterial population among types of fruits could arise from the level of decomposition that occurred during transportation of samples to the laboratory and holding of infested fruits until the larvae reached 3^rd^ instar. Although we performed PERMANOVA analysis to observe difference in bacterial community structure among fruit types, no difference was found in sapodilla between the two sampled sites. While numerous comparisons of fruit types across multiple regions would be required for a detailed analysis, the available data suggest that fruit type may be a greater influence than geographic region in determining fruit microbial communities. In our study, we observed the presence of *Gluconacetobacter* and *Gluconobacter* bacteria both in green apple and quince without any significant difference in number. In contrast, overall bacterial relative abundance was significantly different between these two fruits.

We expected to find a correlation between microbial communities of larvae and their host fruit but, found very little evidence of such relationship. The bacterial community structure in larvae was significantly different not only from the same type of fruit, but to the other types of fruits as well. The only exception was found in sapodilla from Nambour, QLD. Yun *et al*.^41^ found remarkable variation in the bacterial community of insects (e.g. Proteobacteria and Firmicutes) depending on the host environment. Gut bacterial relative abundances may vary with the natural surroundings and the associated oxygen level of the insect (e.g., wood feeding termites^79^). These observations are relevant to our findings. We found that there was no significant variation in the microbiome of *B. tryoni* larvae sampled across different types of fruit. The PERMANOVA pair-wise test did not detect significant variation in the basic bacterial community structure of larvae from different host fruit. Although there was a significant difference in bacterial relative abundance between green apple and quince fruits, no significant variation was present in the larval microbiome from these fruits. This could be related to both fruits being members of the Rosaceae. Previous studies of *B. oleae* and *C. capitata* found very low gut bacterial diversity in larvae collected from the field^24,42^. However, in *C. capitata* bacterial diversity was much higher in pupae and adults than in larvae^42^. Also, larvae of *B. dorsalis*^40^ and *B. carambolae*^36^ have also been reported to have substantially greater bacterial diversity than *B. oleae* and *C. capitata*. Gut bacterial diversity in larvae is generally less than the adults in insects. We found that the microbiome is very simply structured in wild *B. tryoni* larvae. It might be that the bacterial diversity is lower in *B. tryoni* larvae compared to the adults; further study of changes in bacterial diversity through *B. tryoni* metamorphosis will be required to assess this possibility.

Larvae may acquire bacteria from the fruits to develop the gut community. Diet has a significant effect on the gut microbiome composition in other insects^80^, including *Helicoverpa armigera* (Lepidoptera: Noctuidae) (Cotton bollworm)^81^, *Lymantria dispar* L. (Lepidoptera: Lymantriidae) (Gypsy Moth)^1^ and *Heliconius erato* (Butterfly)^82^. We asked whether the bacterial microbiome of *B. tryoni* larvae comes from the host fruit and tested the difference in the bacterial communities of larvae and their host fruits. Based on principal coordination analysis and PERMANOVA tests, we found that the larval bacterial community (mostly from the gut) was significantly different from that of the host fruit. We further observed that the larval microbial community contains common bacteria of *Asaia, Gluconacetobacter, Gluconobacter*, and *Acetobacter* which were present in all larvae and fruit samples. However, the percentage of independent bacteria was significantly higher in larvae compared with each type of fruit (Supplementary Fig. 3). The vast majority of microbial taxa detected in larvae are not found in the fruit. Microbial communities associated with larvae were significantly more diverse than those of fruit. Previous studies have demonstrated that female tephritid fruit flies transmit gut bacteria during oviposition in the areas where the fly previously regurgitated^83^. This suggests that, as has been suggested previously^10,20,39,63^, bacteria are transmitted vertically from the mother to the egg, and then larvae, during oviposition, and remain quite isolated from the surrounding host tissues.

## Conclusion

The present study is the first detailed investigation of relationships between the bacterial ecology of *B. tryoni* larvae and their host fruit in nature. The abundant bacterial taxa in larvae were highly consistent across fruit types and geographic regions despite significant variation in overall bacterial microbiome composition.

## Methods

### Collection of larvae

*Bactrocera tryoni* larvae were collected from infested fruits at various geographic locations in New South Wales (NSW), Victoria (VIC) and Queensland (QLD), Australia (Table 3). All infested fruits were collected from under trees, and most were over-ripe. The fruit types collected included hog plum, sapodilla (from two different locations), pomegranate, green apple and quince (Table 3). The infested fruits were stored on racks in 60 L plastic bins (Award, Australia) that contained 250 g of fine vermiculate (Grade 1, Sage Horticultural, VIC, Australia) in a controlled environment laboratory (25 ± 0.2 °C, 65 ± 3% RH and 11 h: 1 h: 11 h: 1 h light: dusk: dark: dawn photoperiod). Samples of different fruit types and origins were kept separate to prevent cross-contamination. A total of 36 3rd instar *B. tryoni* larvae were collected from each of six replicate fruits from each of the five fruit types (see Table 3). Furthermore, six replicate samples of fruit tissues (fruit flesh) (1~2 mg mass) were collected from the same fruit from which larvae were collected.

**Table 3:** Fruit types and origin for wild *Bactrocera tryoni* larvae collection. A total of six replicate larvae, and fruit flesh samples were collected from each fruit origin.

Geographic location of collection	Fruit source and number of fruits collected	Collection date
Maroochy Research station, Nambour, QLD GPS: Lat 26°38′34.92”, Long 152°56′22.99”	Hog Plum 26 pieces	1/02/17
Daboro Road, Whiteside, QLD, 4503. GPS: Lat 27°14′29.31”, Long 152°55′8.49”	Sapodilla 52 pieces	1/02/17
Maroochy Research station, Nambour, QLD GPS: Lat 26°38′34.92”, Long 152°56′22.99”	Sapodilla 68 pieces	1/02/17
Coomealla, NSW GPS: Lat 34° 5′50.97”, Long 142° 3′7.21”	Pomegranate 37 pieces	5/05/17
St. Germains, Between Tatura and Echuca in Victoria GPS: Lat 36°10′48.86”, Long 145° 8′50.74”	Green Apple 41 pieces	05/05/17
Downer road between Tatura and Toolamba in Victoria GPS: Lat 26°38′34.92”, Long 152°56′22.99”	Quince 52 pieces	05/05/17

### Sample preparation

Upon collection, *B. tryoni* larvae were surface sterilized using 0.5% Tween 80 (Sigma-aldrich, Cat. No. 9005656), 0.5% Bleach (Sodium hypochlorite) (Sigma-Aldrich, Cat. No.7681529) and 80% Ethanol (Sigma-Aldrich, Cat. No. 65175) for 30 s, and rinsed 3 times in 1 M sterile phosphate-buffered saline (1x PBS) again for 30 s, following^39^. The PBS from the 2^nd^ and 3^rd^ washes were kept and 100 μL was spread-plated onto five types of microbial growth medium (de Man, Rogosa and Sharpe Agar, Tryptone Soya Agar, Macconkey Agar, Potato Dextrose Agar and Yeast-dextrose Agar medium) to check the performance of the sterilization method. All plates were incubated at 35 °C for 24 to 48 hr. Post sterilization, whole larvae were crushed using sterile pestles (Fisher Scientific, USA) and stored with Brain Heart Infusion (BHI) broth with 20% Glycerol solution and split into two separate cryovial tubes for COI gene identification and next generation sequencing analysis. All the samples preserved at −80 °C. Flesh from individual fruits was also preserved and stored under the same conditions. All procedures were completed in a sterile environment (Biological Air Clean Bench, safe 2020 1.2, Thermo Scientific, Germany).

### Identification of larvae using mitochondrial Cytochrome Oxidase I (COI) gene

Identification of larvae was confirmed by sequencing the mitochondrial cytochrome oxidase I (COI) gene of all larval samples. DNA was extracted from crushed whole larvae using the Isolate II genomic DNA kit from Bioline (Cat. no. BIO-52066) following the manufacturer’s protocol. DNA extract concentrations were then determined using the Invitrogen™ Qubit® dsDNA High Sensitivity (HS) Assay Kit (Life Technologies, USA). Standard LCO1490 ⁄ HCO2198 primers were used to amplify a 700 bp segment of the CO1 gene^84^. All PCR amplifications were performed in an Eppendorf thermocycler (Mastercycler, epgradient S, Eppendorf, Germany). Each 15 μL reaction was conducted in triplicate and contained 7.5 μL of MyTaq HS PCR master mix (Bioline, USA. Cat No. BIO-25045), 0.60 µL of forward (LCO1490F) and reverse primer (HCO2198R), and 1.5 μL of DNA extract of larval sample. The PCR profile included an initial denaturing step at 95 °C for 2 min, followed by 35 cycles of 94 °C for 30 s, 50 °C for 30 s and 72 °C for 90 s, and a final extension step of 72 °C for 5 min. Amplicons were visualised using electrophoresis on a 1% agarose gel (110 v, 45 min). Amplicons were then sent to the Australian Genomic Research Facility (AGRF) for Sanger sequencing. Sequence data were analysed by Geneious R10.2.3 to confirm that all larvae were *B. tryoni*. In addition to this molecular confirmation, microscopic examination of larval morphological features was carried out prior to DNA extraction^85^. Additional confirmation was gained through stereomicroscopic (Leica MZ6 stereo-microscope, Germany) assessment of adult flies that developed from the larvae remaining in the infested fruits that larval and fruit flesh samples were obtained from^86^.

### Microbiome profiling

DNA extraction of the larvae samples for NGS analysis was completed using DNeasy Power Lyzer Power Soil Kit-100 (Qiagen, Germany) (Cat. no. 12855-100) following the manufacturer’s protocol. DNA extracts were then quantified by Invitrogen™ Qubit® dsDNA High Sensitivity (HS) Assay Kit (Life Technologies, USA). PCR amplification and sequencing were performed by the Australian Genome Research Facility. Briefly, the V1-V3 16S rRNA region was amplified using primers 27 F (5′AGAGTTTGATCMTGGCTCAG-3′) and 519 R (3′ GWATTACCGCGGCKGCTG-5′). Amplification conditions were as in Fouts *et al*.^87^ with slight modifications. Briefly, reactions contained 1X AmpliTaq Gold 360 mastermix (Life Technologies, USA), 0.20 µM of forward and 0.20 µM reverse primers and the total of 25 µL with DNA extract. PCR cycling conditions consisted of denaturation at 95 °C for 7 minutes, 35 cycles of denaturation at 94 °C for 45 s, annealing at 50 °C for 60 s and extension at 72 °C for 60 s, and a final extension of 72 °C for 7 minutes. A secondary PCR was used to adhere sequencing adaptors and indexes to the amplicons. Primerstar max DNA Polymerase used for secondary PCR amplicon generation from Takara Bio inc., Japan (Cat. No. #R045Q). The resulting amplicons were measured by florometry (Invitrogen Picogreen, Thermo Fisher Scientific, Australia) and normalized. The eqimolar amounts of each sample were pooled and quantified qPCR prior to sequencing (Kapa qPCR Library Quantification kit, Roche, Switzerland). The resulting amplicon library was sequenced on the Illumina MiSeq platform (San Diego, CA, USA) with 2 × 300 base pairs paired-end chemistry^88^.

### Sequence data processing

Paired-end reads were assembled by aligning the forward and reverse reads using PEAR (version 0.9.5)^89^. Primers were identified and trimmed. Quality filtering, clustering and taxonomic assignments were achieved using the ‘usearch’ tools^90,91^ and rdp gold database as a reference (Ribosomal database project, https:// rdp.cme.msu.edu). The OTUs with taxonomic assignments to eukaryotic organelles (e.g., choloroplast) were removed from the dataset. We performed rarefaction to 10,000 reads per sample, repeating this 50 times and averaging the counts to obtain a representative rarefaction. This was achieved using an in-house python script. Those samples that had <10,000 reads were deleted. The data were then normalised as the percentage of relative abundance and is referred to as the OTU table (Supplementary Data S1). All the figures of the bacterial relative abundance in *B. tryoni* larval and fruit samples at different taxonomic levels were plotted in Microsoft excel version 16.18.

### Statistical analysis

An OTU table, which contained the number of read counts for each OTU detected for each sample was imported into Primer-E v7^92,93^ for analysis. In brief, all statistical testing was performed on fixed factors associated with fruit host (hog plum, sapodilla (from two different localities), pomegranate, green apple and quince) from which 6 replicates were collected. The DIVERSE function was used to generate univariate biodiversity metrics, including total species, species richness, Pielou’s evenness and Shannon’s and Simpson’s biodiversity indices. Statistical differences between these metrics were assessed by one-way analysis of variance (ANOVA) and Tukey-Kramer post hoc analysis (see Supplementary Table 1) using JMP Statistical Software Version 10.0.0 (SAS Institute, Cary, NC, USA). To observe the taxonomic compositional differences amongst 16 s rRNA communities, the OTU table was first log transformed using Primer-E V7. A Bray-Curtis similarity matrix was derived from this transformed data and a permutational analysis of variance (PERMANOVA) pair wise comparison was conducted to compare all community samples. A p value of <0.05 was considered statistically significant. Ordination plots of these communities were visualised using principal coordinates analysis (PCoA) in Primer-E.

To explore which taxa were driving compositional differences between microbial communities from different groups, genera were investigated for statistically significant differences in their relative abundances. Relative abundance values were first arcsine square root transformed^94^. Subsequent statistical analyses were carried out using an in-house Python script, with the SciPy^95^ and statsmodels^96^ packages. Briefly, a t-test was used to compare relative abundances of genera between total larval microbiome communities and total fruit flesh microbial communities. A Benjamini–Hochberg adjustment to the resultant p value was made to adjust for false-discovery rate errors (FDR). FDR adjusted p-values of <0.05 were considered significant. ANOVA was used to compare genera relative abundances in larval microbiome communities from different fruit type/origin^97^. The resultant p value was again adjusted for FDR, and a posthoc Tukey’s Honest Significant Difference test (Tukey’s HSD) was used to test for significant pair-wise comparisons. This same approach was undertaken to compare genera in fruit flesh microbial communities from different fruit types/origins (Supplementary Data S3). Venn diagram plots for each fruit type were created using R 3.2.2 (R Development Core Team 2017). Percentage of the bacteria present in larvae and fruit samples in Venn diagrams were analysed using in the R package eulerr^98^.

## Supplementary information

Supplementary Information

Dataset 1

Dataset 2

Dataset 3

## Data Availability

All sequences are publically available in NCBI under Bio-project PRJNA528521.
