# Supplementary material for: Next-Generation Sequencing reveals relationship between the larval microbiome and food substrate in the polyphagous Queensland fruit fly
Source: Sci Rep. 2019 Oct 1;9:14292. doi: 10.1038/s41598-019-50602-5 (PMC6773747; doi:10.1038/s41598-019-50602-5)
Supplement: Supplementary file 1 — Supplementary Information [file 41598_2019_50602_MOESM1_ESM.pdf]

Supplementary information

Next-Generation Sequencing reveals relationship between the larval microbiome and food substrate in the polyphagous Queensland fruit fly

Rajib Majumder[1,3*], Brodie Sutcliffe[2], Phillip W Taylor[1] & Toni A Chapman[1,3]

[1]*Department of Biological Sciences, Macquarie University, North Ryde, NSW 2109, Australia*
[2]*Department of Environmental Sciences, Macquarie University, North Ryde, NSW 2109, Australia*
[3]*Biosecurity and Food Safety, NSW Department of Primary Industries, Elizabeth Macarthur Agricultural Institute (EMAI), Menangle, NSW 2567, Australia*

To whom correspondence should be addressed:

Rajib Majumder* (Main Correspondence)
Department of Biological Sciences, Macquarie University,
North Ryde, NSW 2109, Australia
Email: rajib.majumder@mq.edu.au

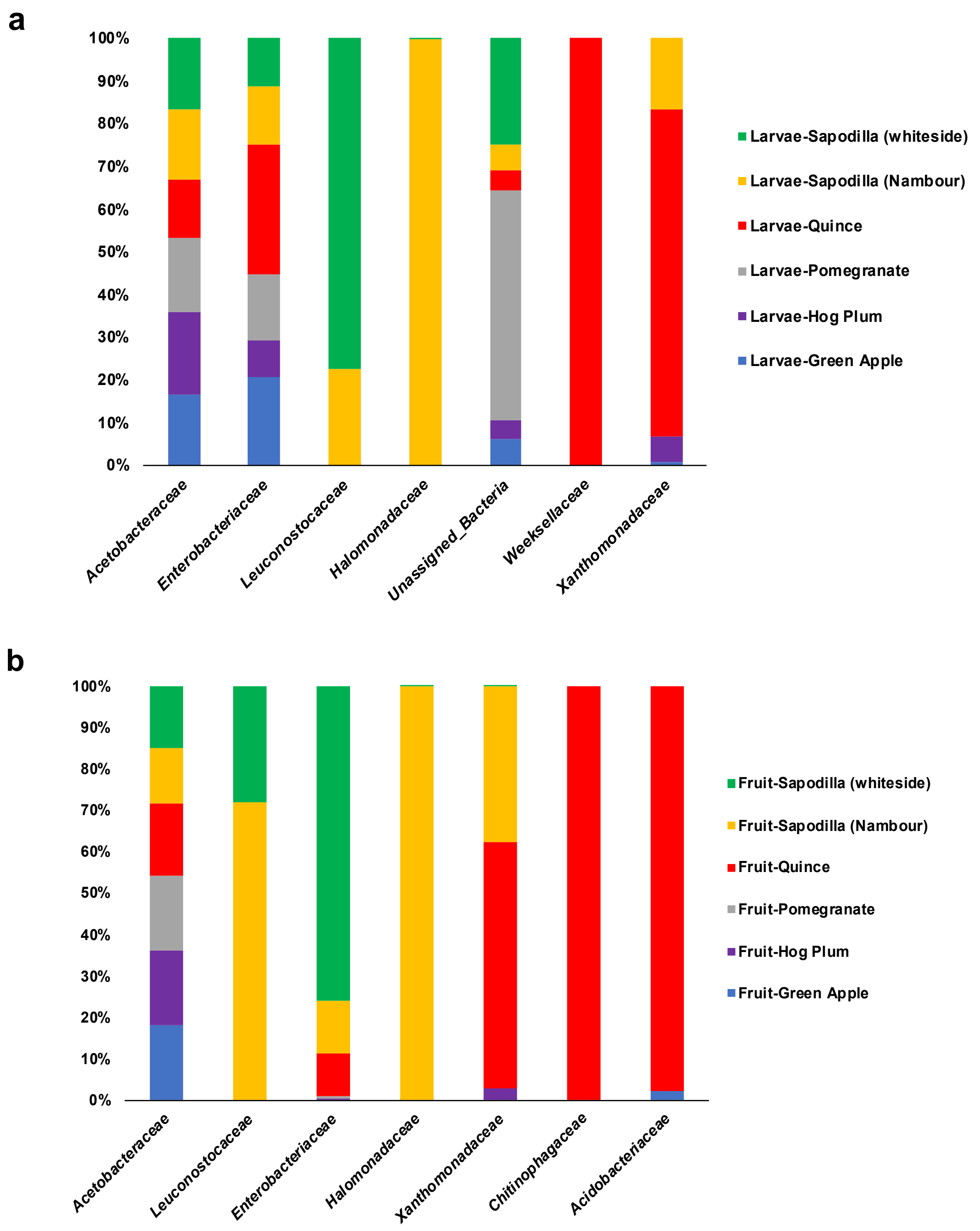

Figure S1. Percentage of mean Relative abundance of the bacteria at the family levels in samples of *B. tryoni* wild larvae and fruit samples; (a) relative abundance of bacteria at the family levels in samples of *B. tryoni* wild larvae obtained from five different types of fruit in the wild. (b) relative abundance of bacteria at the family levels in samples of five different types of the fruit

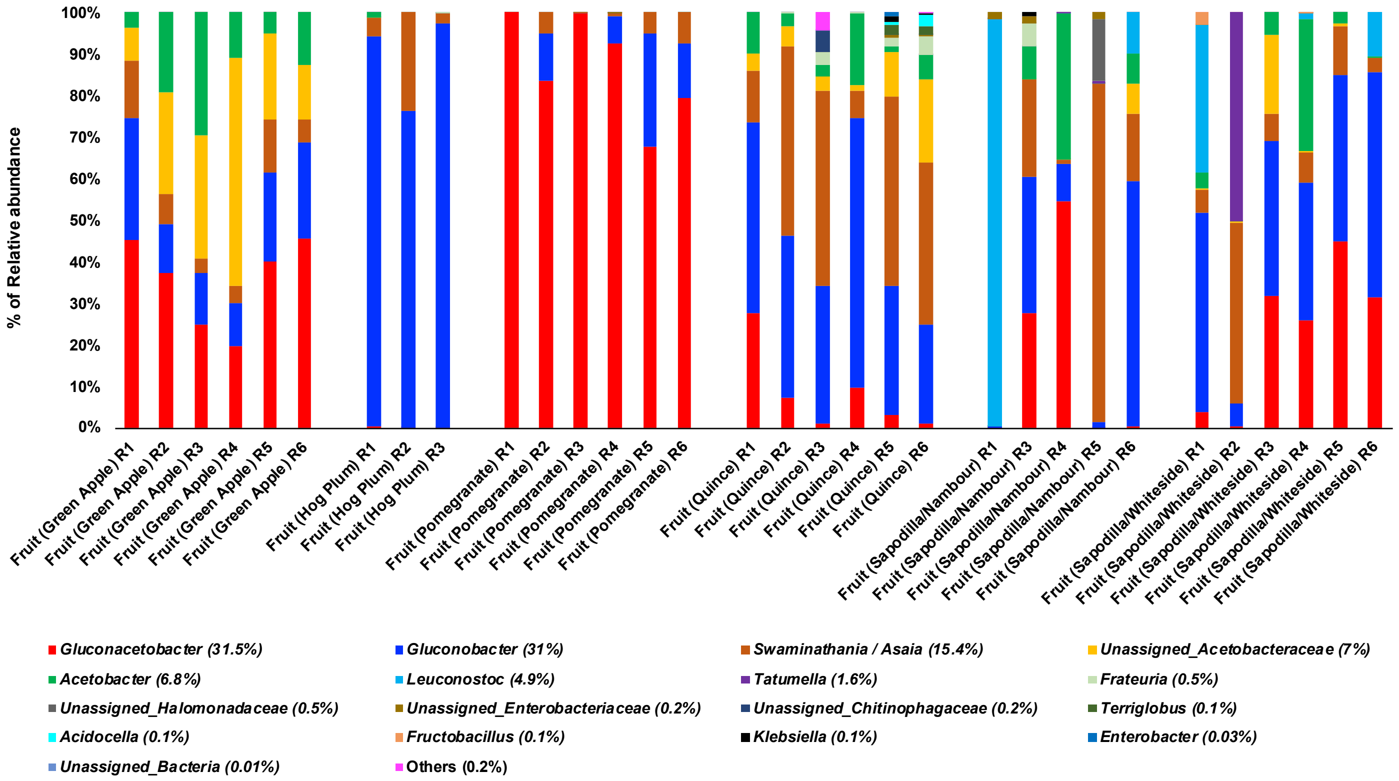

Figure S2. Relative abundance of bacterial taxa of 32 fruit samples. The percentage of relative abundance of four or less than are included in "Others". Each type of fruit sample has 6 identical replicates except Hog plum (3 replicates) and Sapodilla/Nambour (5 replicates). R1 to R6 refers to the replicate number of each fruit.

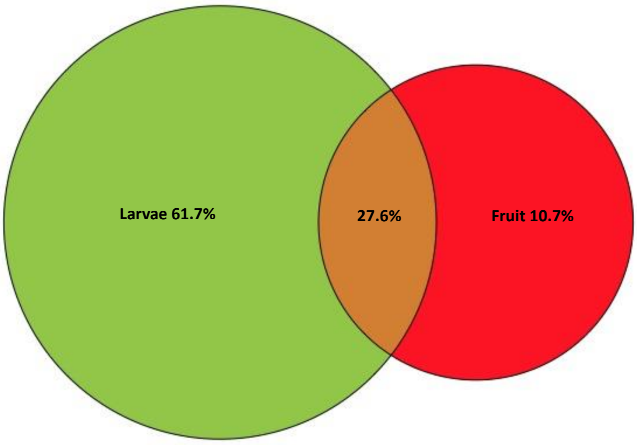

Figure S3. Venn diagram of the average percentage of the bacteria present independently in the larvae, fruit and common in both.

Table S1. Diversity indices of bacterial community composition in the five different fruit and the larval gut of *B. tryoni*. Different letters indicate significant Tukey's post hoc comparisons ($p < .05$)

**Sample name**	**Total Species**	**Species Richness**	**Pielou's evenness**	**Shannon**	**Simpson**
Fruit (Green Apple)	18.5 ± 0.43ab	1.9 ± 0.04ab	0.55 ± 0.01a	0.11 ± 0.04a	0.74 ± 0.17a
Fruit (Hog Plum)	16 ± 2.52ab	1.62 ± 0.27ab	0.13 ± 0.05c	0.34 ± 0.12cd	0.18 ± 0.09cd
Fruit (Pomegranate)	11.66 ± 2.03b	1.15 ± 0.22b	0.15 ± 0.05c	0.39 ± 0.13d	0.21 ± 0.07d
Fruit (Quince)	27.5 ± 3.28ab	2.88 ± 0.35ab	0.46 ± 0.02ab	1.49 ± 0.11ab	0.66 ± 0.02ab
Fruit (Sapodilla/Nambour)	18 ± 1.52ab	1.85 ± 0.16ab	0.32 ± 0.08abc	0.94 ± 0.26abcd	0.47 ± 0.12abcd
Fruit (Sapodilla/Whiteside)	18.33 ± 1.47ab	1.88 ± 0.16ab	0.42 ± 0.02ab	1.24 ± 0.08abc	0.65 ± 0.02abc
Larvae (Green Apple)	26.66 ± 4.27ab	2.78 ± 0.46ab	0.34 ± 0.07abc	1.17 ± 0.24abcd	0.54 ± 0.11abcd
Larvae (Hog Plum)	36 ± 4.83a	3.8 ± 0.52a	0.16 ± 0.04c	0.6 ± 0.16cd	0.33 ± 0.09bcd
Larvae (Pomegranate)	32.33 ± 3.05a	3.41 ± 0.33a	0.28 ± 0.02bc	0.93 ± 0.06abcd	0.45 ± 0.04abcd
Larvae (Quince)	33 ± 3.99a	3.51 ± 0.43a	0.25 ± 0.06bc	0.86 ± 0.21abcd	0.44 ± 0.1abcd
Larvae (Sapodilla/Nambour)	36.83 ± 9.83a	3.89 ± 1.06a	0.22 ± 0.03bc	0.79 ± 0.12bcd	0.39 ± 0.06abcd
Larvae (Sapodilla/Whiteside)	34.5 ± 4.23a	3.64 ± 0.45a	0.26 ± 0.06bc	0.94 ± 0.23abcd	0.45 ± 0.1abcd

Supplementary dataset:

Supplementary dataset S1: OTU table

Supplementary dataset S2: Mapping file

Supplementary dataset S3: FDR Correction
